# The Bile Acid–Diet–Microbiome Axis in *Clostridioides difficile* Infection

**DOI:** 10.3390/nu18172872

**Published:** 2026-09-02

**Authors:** Felicia Trofin, Elena Roxana Buzila, Irina Roxana Iancu, Cristina Gabriela Tuchilus, Oana-Raluca Temneanu, Luminita Smaranda Iancu, Madalina Alexandra Vlad, Dana-Teodora Anton-Păduraru, Ioana Armasu, Aida Corina Badescu, Laura Gisca, Olivia Simona Dorneanu

**Affiliations:** 1 Grigore T. Popa University of Medicine and Pharmacy Iasi, 700115 Iasi, Romania; elena-roxana.buzila@umfiasi.ro (E.R.B.); cristina.tuchilus@umfiasi.ro (C.G.T.); temneanu.oana@umfiasi.ro (O.-R.T.); luminita.iancu@umfiasi.ro (L.S.I.); madalina.vlad@umfiasi.ro (M.A.V.); dana.anton@umfiasi.ro (D.-T.A.-P.); ioana.armasu@umfiasi.ro (I.A.); aida.badescu@umfiasi.ro (A.C.B.); olivia.dorneanu@umfiasi.ro (O.S.D.); 2 Sf. Spiridon County Clinical Emergency Hospital Iasi, 700111 Iasi, Romania; 3 Iasi Regional Center for Public Health, National Institute of Public Health, 700465 Iasi, Romania; 4 Sf. Maria Children Emergency Hospital, 700309 Iasi, Romania; 5 Sf. Parascheva Clinical Hospital of Infectious Diseases, 700116 Iasi, Romania; 6 Kendrick School, Reading RG1 5BN, UK; 20lgisca@kendrick.reading.sch.uk

**Keywords:** *Clostridioides difficile*, bile acids, microbiome, diet, dysbiosis, fecal microbiota transplantation, CDI

## Abstract

*Clostridioides difficile* infection (CDI) remains a major cause of antibiotic-associated diarrhea, with recurrence largely driven by disruption of the gut microbiota and impaired colonization resistance. Bile acids have emerged as key mediators in this process, as primary conjugated bile acids promote *C. difficile* spore germination, whereas microbiota-derived secondary bile acids can inhibit germination, vegetative growth, and toxin activity. Diet further modulates CDI susceptibility by shaping microbial composition, short-chain fatty acid production, bile acid transformation, epithelial barrier integrity, and intestinal inflammation. Western-style diets, and low fiber intake, may favor dysbiosis and a bile acid profile permissive to CDI, while fiber-rich and Mediterranean-type dietary patterns may support beneficial anaerobes, microbial metabolites, and mucosal resilience. This narrative review summarizes current evidence on the bile acid–diet–microbiome axis in CDI pathogenesis, recurrence, and therapy. It also discusses standard treatment limitations, microbiome-based therapeutics, dietary interventions, and emerging bile acid-targeted strategies. Understanding this axis may support future precision approaches aimed not only at suppressing *C. difficile*, but also at restoring microbiota function and durable colonization resistance.

## 1. Introduction

*Clostridioides difficile* infection (CDI) is the leading identifiable cause of antibiotic-associated diarrhea, accounting for approximately 15–25% of cases [1,2]. Originally recognized as a predominantly healthcare-associated pathogen, CDI has undergone a notable epidemiological shift over the past two decades, with increasing incidence in community settings and among populations previously considered low risk, including healthy adults, children, and individuals without recent antibiotic exposure or healthcare contact. The global burden of CDI has risen substantially, accompanied by increased morbidity, mortality, and healthcare costs [1,2,3,4,5]. This trend has been partly attributed to the emergence of hypervirulent strains, particularly PCR ribotype 027, which have been associated with more severe disease outcomes [6]. Older adults remain disproportionately affected, with the highest incidence and mortality observed in individuals aged 65 years and older [1,2,3,4,5,6]. In the EU/EEA, CDI carries a substantial disease burden, ranking fourth among surveyed infectious diseases in disability-adjusted life years during 2009–2013. An estimated 189,526 healthcare-associated CDI cases occurred annually in acute care hospitals during 2016–2017, contributing to approximately 7419 deaths. Between 2018 and 2020, healthcare-associated cases consistently represented 59–63% of all CDI, with community-associated and recurrent infections comprising the remainder. Tertiary care hospitals report particularly high incidence, likely reflecting greater patient turnover and case complexity [7,8].

The gut microbiota and bile acid pool are closely interconnected and together contribute to intestinal homeostasis, metabolism, and immune regulation. Primary bile acids are transformed by intestinal microorganisms into a diverse range of secondary bile acids through enzymatic reactions including deconjugation, dehydroxylation, and epimerization. These processes are mediated by bacterial taxa such as *Clostridium*, *Bacteroides*, *Lactobacillus*, *Bifidobacterium*, and *Enterococcus*. In turn, bile acids influence the structure of the gut microbial community through their antimicrobial activity, selectively inhibiting susceptible organisms while favoring bile acid–metabolizing species. These pathways regulate epithelial barrier integrity, inflammatory responses, glucose metabolism, and T-cell differentiation [9,10,11,12].

Diet is increasingly recognized as a modifiable determinant of gut microbial structure and function, with direct implications for bile acid metabolism, short-chain fatty acid production, epithelial barrier integrity, and inflammatory tone. In CDI, these diet-sensitive pathways are particularly relevant because antibiotic-induced dysbiosis alters the balance between germination-promoting primary bile acids and inhibitory secondary bile acids. Thus, nutritional patterns may influence not only the recovery of the gut microbiota after antibiotic exposure, but also the metabolic environment that determines *C. difficile* germination, growth, toxin activity, and recurrence [13].

### 1.1. Aim

**Figure 1:**
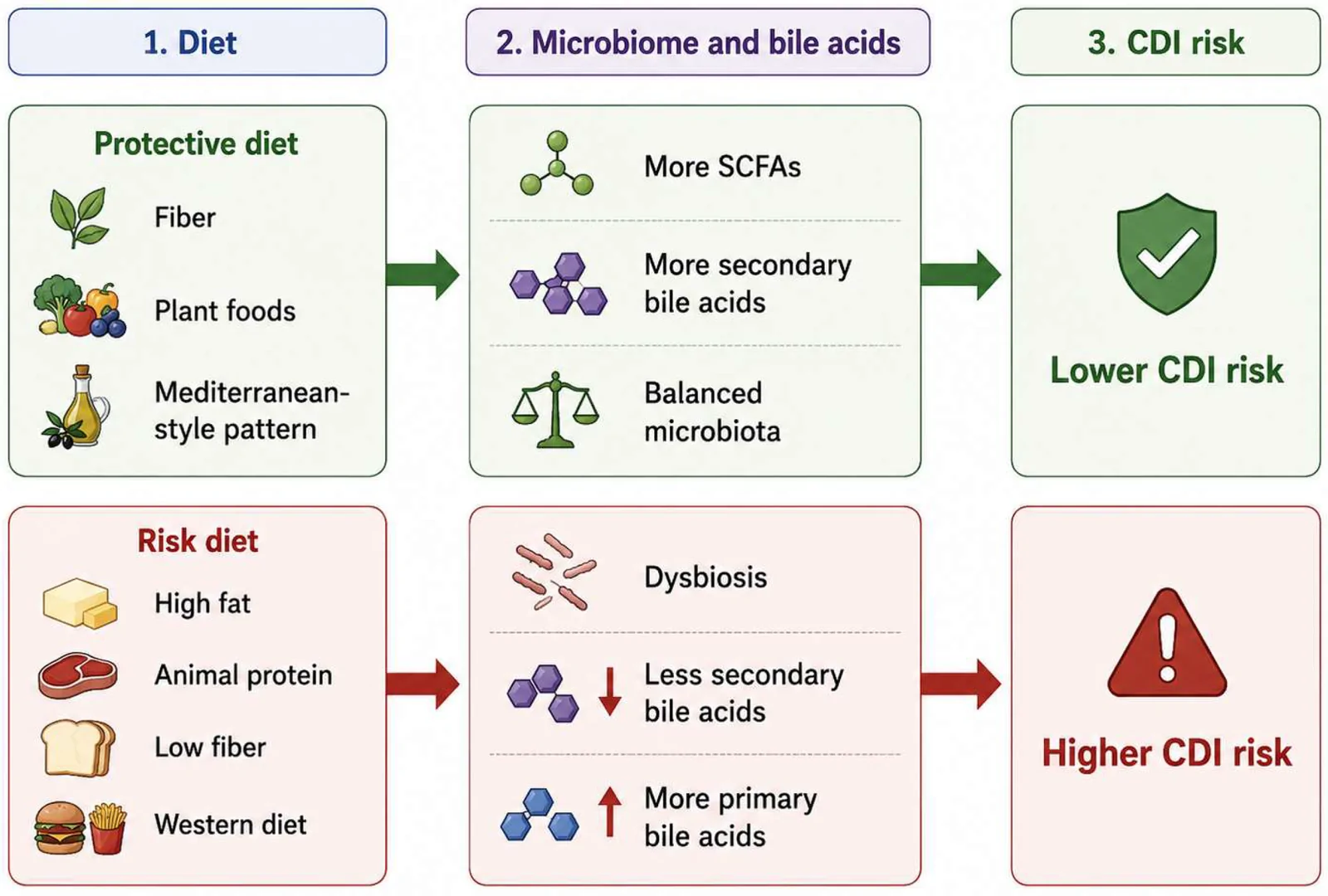
Interactions between diet, the gut microbiome, and bile acid metabolism in shaping susceptibility to *Clostridioides difficile* infection.

This narrative review aims to examine the complex interplay between bile acid metabolism, dietary factors, and the gut microbiome in the pathogenesis of CDI. Growing evidence indicates that disruptions in the bile acid–microbiome axis play a central role in the loss of colonization resistance, facilitating *C. difficile* germination, growth, toxin production, and disease recurrence. At the same time, diet has emerged as a key modulator of both microbial composition and bile acid metabolism, with potential implications for CDI susceptibility and clinical outcomes. This review summarizes current knowledge on the mechanisms linking bile acids, the intestinal microbiota, and host immunity, with particular emphasis on the influence of dietary patterns and nutritional interventions. In addition, it highlights emerging therapeutic opportunities that target the bile acid–diet–microbiome axis and discusses their potential role in the prevention and management of CDI (Figure 1).

### 1.2. Literature Search Strategy and Study Selection

This narrative review was based on a targeted literature search focused on the relationship between diet, bile acid metabolism, the gut microbiome, and *Clostridioides difficile* infection (CDI). The search was performed in PubMed and Google Scholar using combinations of the following terms: “*Clostridioides difficile*”, “CDI”, “bile acids”, “gut microbiota”, “diet”, “nutrition”, “dysbiosis”, “short-chain fatty acids”, and “therapy”. Boolean operators were used to combine these terms and to identify publications relevant to the diet–bile acid–microbiome axis in CDI.

After duplicate records were removed, titles and abstracts were screened for relevance to the objectives of the review. Publications were considered for inclusion when they addressed at least one of the following topics: bile acid metabolism in CDI, microbiota-mediated colonization resistance, diet-induced modulation of the gut microbiome, short-chain fatty acid production, antibiotic-induced dysbiosis, recurrent CDI, dietary or nutritional factors influencing CDI susceptibility, microbiome-based therapies, fecal microbiota transplantation, bile acid-targeted interventions, or the role of artificial nutrition in critically ill patients.

Full-text articles were then assessed when the title and abstract indicated potential relevance. Studies were excluded if they were only indirectly related to CDI, focused exclusively on unrelated hepatobiliary or gastrointestinal conditions, did not address diet, bile acids, microbiome-related mechanisms, or therapeutic implications, or if the full text was not available. Articles were also excluded when their scope was not aligned with the central question of the review or when the available information was insufficient to support inclusion in the qualitative synthesis. Additional relevant publications were identified by manually screening the reference lists of selected articles.

A total of 119 publications were included in the final synthesis. The selected studies were analyzed qualitatively, with attention to methodological quality, mechanistic relevance, clinical applicability, and their contribution to understanding how the diet–bile acid–microbiome axis influences CDI susceptibility, pathogenesis, recurrence, and therapeutic modulation. As this manuscript was designed as a narrative review, rather than a systematic review or meta-analysis, no formal quantitative synthesis or systematic risk-of-bias assessment was performed. Nevertheless, the literature selection was guided by predefined thematic criteria to ensure that the included references were relevant to the scope and objectives of the review.

## 2. Biology and Metabolism of Bile Acids

### 2.1. Synthesis and Enterohepatic Circulation

Bile acids are synthesized from cholesterol in the liver through the classical and alternative pathways. These pathways generate the primary bile acids cholic acid (CA) and chenodeoxycholic acid (CDCA), which are subsequently conjugated with glycine or taurine before being secreted into bile [14,15,16,17,18]. This conjugation increases their solubility and facilitates their release into the intestinal lumen.

In the small intestine, bile acids act as amphipathic molecules that contribute to the digestion and absorption of dietary lipids and fat-soluble vitamins. Most bile acids are then reabsorbed in the terminal ileum and returned to the liver through the enterohepatic circulation, allowing repeated reuse of the bile acid pool [11,16,17,18,19].

This cycle is relevant to CDI because the amount and composition of bile acids entering the intestine are influenced by hepatic synthesis, intestinal reabsorption, diet, and microbial metabolism. Changes in this system may alter the balance between primary bile acids, which can promote *C. difficile* spore germination, and secondary bile acids, which are largely produced by the gut microbiota and may inhibit *C. difficile* growth and toxin activity.

### 2.2. Microbial Transformation of Bile Acids

During intestinal transit, the gut microbiota modifies bile acids through processes such as deconjugation, reconjugation or amino acid exchange, dehydrogenation, dehydroxylation, and epimerization, thereby increasing their diversity and altering their physicochemical properties [11]. These transformations convert primary bile acids into secondary bile acids, including deoxycholic acid (DCA), ursodeoxycholic acid (UDCA), and lithocholic acid (LCA) [16,17,18]. The deconjugation of primary bile acids into secondary bile acids by the gut microbiome occurs in several steps involving deconjugation by bile salt hydrolase (BSH), followed by 7α-dehydroxylation [10,20].

The abundance and diversity of bacteria that carry out the various transformations of bile acids varies enormously. The oxidation of hydroxyl groups in bile acids is a common activity in intestinal microorganisms and is catalyzed by hydroxysteroid dehydrogenases (HSDHs) [21].

7α-Dehydroxylation is one of the most important transformations of bile acids, generating secondary bile acids, including DCA and LCA. This multistep biochemical pathway, encoded by the bai (‘bile acid inducible’) gene operon, is restricted to a phylogenetically restricted group of bacterial species belonging to *Clostridium* cluster XIV and has been most extensively studied in the species *Clostridium scindens* and *Clostridium hylemonae*. The transformation of host-produced conjugated primary bile acids into DCA and LCA is an example of interspecific microbial co-metabolism [22].

These microbial transformations provide a mechanistic link between diet and CDI, since dietary substrates that support anaerobic, bile acid–modifying bacteria may favor the production of secondary bile acids with inhibitory effects against *C. difficile*.

### 2.3. Bile Acids as Signaling Molecules

Beyond their role in lipid digestion, bile acids also act as signaling molecules that regulate metabolic, epithelial, and immune functions. These effects are mediated mainly through receptors such as the farnesoid X receptor (FXR), Takeda G protein-coupled receptor 5 (TGR5), pregnane X receptor (PXR), vitamin D receptor (VDR), and RORγt-related pathways [11,20,23,24].

FXR is expressed in the intestine and liver and contributes to the regulation of bile acid synthesis through the intestinal FXR–FGF15/19–hepatic CYP7A1 feedback pathway. Through this mechanism, bile acid signaling helps control the size and composition of the bile acid pool. TGR5 and other bile acid-sensitive pathways also influence glucose metabolism, epithelial barrier function, intestinal motility, and inflammatory responses [10,11,20,24,25].

These signaling functions are relevant to the diet–microbiome–bile acid axis because dietary patterns and microbial metabolism can modify bile acid composition and receptor activation. For example, fiber-rich diets, dietary polyphenols, and microbiota-derived secondary bile acids may influence inflammatory tone, epithelial resilience, and colonization resistance. In CDI, these mechanisms may contribute to whether the intestinal environment remains protective or becomes permissive to *C. difficile* germination, growth, toxin activity, and recurrence.

## 3. Bile Acids in *Clostridioides difficile* Pathogenesis

### 3.1. Lifecycle of C. difficile

Sporulation and germination are central to the persistence, transmission, and pathogenesis of *C. difficile*. Although the precise signals that initiate sporulation remain incompletely defined, nutrient limitation, quorum sensing, and other environmental stressors appear to contribute to this process. Sporulation is coordinated by the master regulator Spo0A, together with nutrient-sensing regulators such as CodY and CcpA, which link metabolic status to spore formation and virulence. The resulting spores are highly resistant structures composed of a dehydrated core, protective cortex, coat layers, and, in some strains, an exosporium, allowing survival under adverse environmental conditions. In the host intestine, germination represents the first step toward active infection and is driven mainly by host-derived bile acids, particularly taurocholate and other CA derivatives, together with amino acid co-germinants such as glycine. Unlike many other spore-forming bacteria, *C. difficile* lacks classical Ger-type germinant receptors and instead relies on Csp-family proteins, especially CspC, to sense bile acid signals and activate downstream cortex degradation through SleC [26].

Secondary bile acids can inhibit spore germination, vegetative growth, and toxin activity, although the magnitude of these effects varies by bile acid species. Thus, the balance between germination-promoting primary bile acids, inhibitory secondary bile acids, available amino acids, and the surrounding microbiota strongly influences whether *C. difficile* remains dormant, colonizes asymptomatically, or progresses to symptomatic infection [27,28].

The dependence of *C. difficile* germination on bile acids and amino acid co-germinants provides a direct rationale for considering how diet and microbial metabolism shape the intestinal signals that initiate infection.

### 3.2. Primary Bile Acids and Spore Germination

Bile acids are key environmental signals that regulate *C. difficile* spore germination and subsequent disease development. Unlike many spore-forming bacteria, *C. difficile* responds primarily to host-derived bile acids rather than classical nutrient germinants. Primary bile acids, especially taurocholate and other cholate derivatives, promote germination in the presence of amino acid co-germinants such as glycine, whereas chenodeoxycholate and several microbiota-derived secondary bile acids inhibit germination, vegetative growth, or toxin activity. In a healthy gut, commensal bacteria convert primary bile acids into secondary bile acids, helping maintain colonization resistance. Antibiotic-induced dysbiosis disrupts this conversion, increasing the relative abundance of germination-promoting bile acids and reducing inhibitory secondary bile acids, thereby creating conditions favorable for *C. difficile* outgrowth (Table 1) [29,30,31,32,33].

Because dietary patterns can influence bile acid secretion and microbial conversion of primary bile acids, nutrition may contribute to the balance between germination-promoting and germination-inhibiting signals in the gut.

### 3.3. Secondary Bile Acids as Inhibitors

Secondary bile acids are key microbiota-derived metabolites that contribute to colonization resistance against *C. difficile*. In contrast to taurocholate and other primary bile acids that promote spore germination, several secondary bile acids, including DCA, LCA, UDCA, and their microbial epimers, can inhibit germination, vegetative growth, toxin expression, or toxin activity. These inhibitory effects vary according to bile acid structure and *C. difficile* strain, but they collectively demonstrate that secondary bile acids can interfere with multiple stages of the pathogen life cycle. Their production depends on specific microbial functions, including BSH activity and 7α-dehydroxylation by taxa such as *C. scindens* and related anaerobes. Antibiotic-induced loss of these bile acid–modifying bacteria shifts the intestinal bile acid pool toward germination-promoting primary bile acids and reduces colonization resistance. Conversely, restoration of secondary bile acid metabolism after fecal microbiota transplantation or through bile acid–modifying commensals has been associated with reduced recurrence and improved protection against CDI [34,35,36,37,38,39].

This inhibitory role of secondary bile acids supports the nutritional relevance of the pathway, as diets that promote microbial diversity and anaerobic bile acid metabolism may help maintain a bile acid profile less favorable to CDI.

### 3.4. Antibiotic-Induced Disruption of Bile Acid Metabolism

Antibiotic exposure disrupts bile acid metabolism by depleting key members of the gut microbiota involved in bile acid transformation, particularly taxa capable of bile salt deconjugation and 7α-dehydroxylation. This loss reduces the production and the activity of secondary bile acids such as DCA and LCA. At the same time, antibiotics favor the accumulation of primary bile acids, including taurocholate and CA derivatives. Experimental and clinical data consistently show that CDI susceptibility and recurrence are associated with reduced microbial diversity, depletion of bile acid–transforming bacteria such as *C. scindens*, and a shift toward germination-promoting bile acid pools. Conversely, restoration of secondary bile acid metabolism after fecal microbiota transplantation or through bile acid–modifying commensals is linked to recovery of colonization resistance. These findings highlight antibiotic-induced bile acid dysmetabolism as a central mechanism connecting microbiota disruption with CDI pathogenesis and recurrence [34,35,36,37,38,39].

In this context, diet may become particularly important after antibiotic exposure, when the availability of fermentable substrates can influence microbial recovery, secondary bile acid production, and restoration of colonization resistance.

### 3.5. Key Microbial Taxa Involved

Key microbial taxa involved in bile acid metabolism influence *C. difficile* infection by shaping the balance between germination-promoting primary bile acids and inhibitory secondary bile acids. BSH activity, which deconjugates primary bile acids, is widely distributed among gut bacteria, particularly within *Bacteroides*, *Blautia*, *Lactobacillus*, *Bifidobacterium*, *Enterococcus*, *Clostridium*, *Roseburia*, and members of the *Firmicutes*, *Bacteroidetes*, and *Actinobacteria* phyla. In contrast, 7α-dehydroxylation is restricted to a smaller group of anaerobes, mainly *Clostridium* clusters XIVa and XI, including *C. scindens* and *Clostridium hiranonis*, as well as selected *Eubacterium*, *Peptostreptococcus*, *Ruminococcaceae*, and *Lachnospiraceae* members. Loss of these bile acid–transforming taxa after antibiotic exposure reduces secondary bile acid production and favors bile acid profiles that promote *C. difficile* germination and outgrowth. Conversely, the presence or restoration of organisms such as *C. scindens* has been associated with increased resistance to CDI, partly through secondary bile acid production and possibly through additional antimicrobial metabolites. These findings identify bile acid–modifying bacteria as central mediators of colonization resistance and as potential targets for microbiome-based CDI prevention strategies [11,38,39,40,41,42].

These taxa are diet-sensitive components of the gut ecosystem, suggesting that nutritional strategies capable of supporting anaerobic and bile acid-transforming bacteria may contribute to CDI prevention or recovery.

### 3.6. Animal and Human Evidence

Evidence from both animal models and human studies supports a central role for bile acid metabolism in susceptibility to CDI and disease recurrence. In murine models, antibiotic treatment consistently disrupts the gut microbiota, leading to depletion of bile acid–transforming bacteria, reduced concentrations of secondary bile acids, and accumulation of primary bile acids that promote spore germination and vegetative outgrowth [35,43]. Conversely, resistance to CDI is associated with the presence of secondary bile acids such as DCA, LCA, UDCA, and related derivatives, which inhibit different stages of the *C. difficile* life cycle [44]. Restoration of bile acid metabolism through fecal microbiota transplantation (FMT) or administration of bile acid–modifying bacteria, particularly *C. scindens*, has been shown to re-establish colonization resistance and reduce disease severity in experimental models [45].

Human metabolomic studies have reported similar findings, demonstrating that patients with primary or recurrent CDI exhibit increased levels of primary bile acids and reduced concentrations of secondary bile acids compared with healthy individuals. Importantly, successful FMT is associated with recovery of secondary bile acid production, particularly deoxycholic and LCA, together with restoration of microbial functions involved in bile acid transformation [42,46]. Longitudinal studies further indicate that persistent depletion of secondary bile acids and bile acid-modifying bacteria correlates with an increased risk of CDI recurrence, whereas recovery of these metabolic pathways is associated with sustained clinical remission. Collectively, these observations identify alterations in the bile acid metabolome as both a marker and a mechanistic contributor to CDI susceptibility, persistence, and recurrence [37,42,47,48].

Taken together, these experimental and clinical observations provide the biological basis for dietary approaches aimed at restoring microbial metabolism, secondary bile acid production, and resistance to CDI recurrence.

## 4. Diet as a Modulator of Bile Acids and *Clostridioides difficile* Infection Susceptibility

### 4.1. Diet–Microbiome–Bile Acid Interactions

Dietary composition is a major determinant of gut microbiome structure and bile acid metabolism, with important implications for CDI (Table 2). Diets rich in fiber support microbial diversity and favor the recovery of commensal taxa after antibiotic exposure, including bacteria with BSH activity and plant glycan–degrading capacity, thereby contributing to a bile acid profile less permissive to *C. difficile* germination. In contrast, low-fiber or fiber-free diets may prolong CDI susceptibility by delaying microbiota recovery and maintaining elevated levels of conjugated primary bile acids, particularly taurocholate, which promotes spore germination. High-fat and ketogenic dietary patterns can also reshape bile acid metabolism by increasing bile acid synthesis and altering the abundance and activity of bile acid–modifying bacteria, including BSH-producing taxa from *Bacteroidetes* and *Firmicutes*. These changes may influence not only bile acid composition but also immune regulation, as microbial bile acid derivatives such as 3-oxo-LCA can modulate inflammatory T-cell responses. Experimental studies further indicate that the effects of dietary fiber are not uniform, with different substrates such as pectin, inulin, cellulose, and mixed microbiota-accessible carbohydrates producing variable effects on CDI outcomes. Overall, current evidence suggests that diet influences CDI risk through integrated effects on microbiota recovery, bile acid transformation, colonization resistance, and host inflammatory pathways [49,50,51,52,53].

### 4.2. High-Fat Diets

High-fat diets substantially influence bile acid metabolism by increasing the demand for lipid emulsification and stimulating hepatic bile acid synthesis and intestinal bile acid secretion. These changes expand and reshape the bile acid pool, while also exerting selective pressure on the gut microbiota. As a result, high-fat diets may favor bile-tolerant and bile acid–transforming bacteria, including taxa with BSH, hydroxysteroid dehydrogenase, and 7α-dehydroxylation activity. Studies in animal models and humans show that dietary fat quantity and fatty acid composition can modify the relative abundance of primary, conjugated, and secondary bile acids, with downstream effects on Farnesoid-X receptor-fibroblast growth factor (FXR–FGF15/19) and Takeda G Protein-Coupled Receptor 5 (TGR5) signaling, lipid absorption, glucose metabolism, and inflammatory responses [40,62,63,64,65,66].

In the context of CDI, these diet-induced shifts may be relevant because conjugated primary bile acids, particularly taurocholate, promote spore germination, whereas several secondary bile acids, including DCA and LCA, inhibit vegetative growth and toxin activity. However, the consequences of high-fat diets appear to depend on fat source, host metabolic status, enterohepatic circulation, and the baseline microbiota, indicating that their effects on CDI risk are likely context-dependent rather than uniform [40,62,66].

High-fat diets, particularly when combined with low fiber intake, appear to worsen CDI outcomes in experimental models. Western-type high-fat/low-fiber diets have been associated with greater antibiotic-induced disruption of the gut microbiota, delayed recovery of protective anaerobic communities, reduced production of secondary bile acids such as deoxycholic and LCA, and increased levels of conjugated primary bile acids, including taurocholate, which promotes *C. difficile* spore germination. This creates a bile acid environment more favorable to colonization, vegetative growth, and disease progression. Several animal studies report higher *C. difficile* burden, impaired bacterial clearance, increased diarrhea, weight loss, intestinal injury, toxin-associated pathology, and mortality in mice or hamsters fed high-fat or Western-style diets. These effects may reflect a combined “double hit,” in which dietary fat increases the delivery of primary bile acids to the intestine, while antibiotic-induced dysbiosis limits their microbial conversion into inhibitory secondary bile acids. However, the effect of dietary fat is not uniform, as CDI severity also depends on fiber content, protein intake, fat source, host metabolic status, baseline microbiota, antibiotic regimen, and the *C. difficile* strain used. Thus, high-fat diets may contribute to CDI susceptibility and severity mainly by reshaping the microbiome–bile acid axis, but their impact should be interpreted individually [13,58,59,67].

These findings suggest that the impact of dietary fat on CDI should be interpreted through its combined effects on bile acid secretion, microbiota disruption, secondary bile acid depletion, and inflammatory responses.

### 4.3. Fiber and Microbiota-Accessible Carbohydrates

Dietary fiber and microbiota-accessible carbohydrates (MACs) are important substrates that support gut microbial metabolism and contribute to colonization resistance against *C. difficile*. After escaping digestion in the small intestine, MACs reach the colon, where anaerobic bacteria ferment them into short-chain fatty acids (SCFAs), mainly acetate, propionate, and butyrate. These metabolites strengthen epithelial barrier function, support mucin production, lower luminal pH, regulate inflammation, and may directly inhibit *C. difficile* growth in a concentration-dependent manner. Fiber-rich diets also favor the recovery of beneficial strict anaerobes, including SCFA-producing and bile acid–modifying taxa such as members of *Lachnospiraceae* and *Ruminococcaceae*. In contrast, low-fiber or MAC-deficient diets impair microbiota recovery after antibiotic exposure, reduce protective metabolites, promote dysbiosis, and may facilitate persistent *C. difficile* carriage or more severe disease. Experimental studies suggest that reintroduction of MACs or selected fibers, including inulin, pectin, fructooligosaccharides, xanthan gum, and resistant starches, can improve microbial resilience, reduce *C. difficile* burden, and enhance bacterial clearance, although effects vary according to fiber type and baseline microbiota composition. Overall, fiber and MACs modulate CDI risk by promoting anaerobic microbial metabolism, SCFA production, epithelial resilience, and restoration of colonization resistance after microbiota disruption [13,59,68,69,70].

Through these mechanisms, dietary fiber provides one of the clearest nutritional links to CDI, as it may support microbial recovery, SCFA production, bile acid transformation, and durable colonization resistance after antibiotics.

### 4.4. Protein-Rich and Western Diets

Protein-rich and Western-style diets may contribute to CDI susceptibility and severity by disrupting the microbiome–metabolite environment that normally supports colonization resistance. These diets are typically rich in animal protein, saturated fat, refined sugars, and ultra-processed foods, while being poor in dietary fiber and microbiota-accessible carbohydrates. Experimental studies suggest that Western-type high-fat/low-fiber diets intensify antibiotic-induced microbiota disruption, reduce beneficial anaerobic and butyrate-producing taxa, lower protective metabolites such as short-chain fatty acids and secondary bile acids, and increase bile acid profiles that favor *C. difficile* germination and growth. In murine models, high-fat/low-fiber or high-fat/high-protein diets have been associated with impaired *C. difficile* clearance, increased diarrhea, weight loss, intestinal injury, toxin-related pathology, and higher mortality. High protein intake may further support *C. difficile* expansion by increasing the availability of amino acids, including proline, which can be used as an energy source by the pathogen, while also promoting microbial metabolites linked to epithelial injury and inflammation. Human studies remain limited, but patients with CDI, particularly recurrent CDI, often show reduced microbial richness, depletion of butyrate-producing taxa such as *Lachnospiraceae* and *Ruminococcaceae*, expansion of *Enterobacteriaceae*, and reduced capacity for bile acid transformation. Overall, Western and protein-rich dietary patterns may worsen CDI risk and outcomes by combining low fiber availability, impaired SCFA production, altered bile acid metabolism, and a pro-inflammatory intestinal milieu, although their effects depend on macronutrient composition, baseline microbiota, antibiotic exposure, host factors, and the *C. difficile* strain involved (Figure 1) [13,69,71].

Therefore, Western-type dietary patterns may influence CDI not through a single nutrient effect, but through the combined loss of fermentable substrates, altered amino acid availability, impaired bile acid transformation, and a more inflammatory intestinal environment.

### 4.5. Mediterranean Diet and Protective Dietary Patterns

The Mediterranean diet is characterized by a high intake of plant-based foods, including vegetables, fruits, legumes, whole grains, nuts, and seeds, with extra-virgin olive oil as the main source of fat, moderate consumption of fish and fermented dairy products, and limited intake of red meat, refined sugars, and saturated fats. This dietary pattern provides abundant fiber, microbiota-accessible carbohydrates, polyphenols, monounsaturated fatty acids, and omega-3 polyunsaturated fatty acids, all of which can influence gut microbial composition and metabolic activity. By promoting beneficial commensal bacteria and increasing the production of short-chain fatty acids such as acetate, propionate, and butyrate, the Mediterranean diet supports epithelial barrier integrity, mucin production, immune regulation, and anti-inflammatory responses. Its high polyphenol content, particularly from extra-virgin olive oil, fruits, vegetables, legumes, and wine consumed in moderation, may further contribute antioxidant, antimicrobial, and immunomodulatory effects. In contrast to Western dietary patterns, the Mediterranean diet may help preserve microbial diversity, favor SCFA-producing taxa, and reduce low-grade intestinal inflammation [72,73].

**Figure 2:**
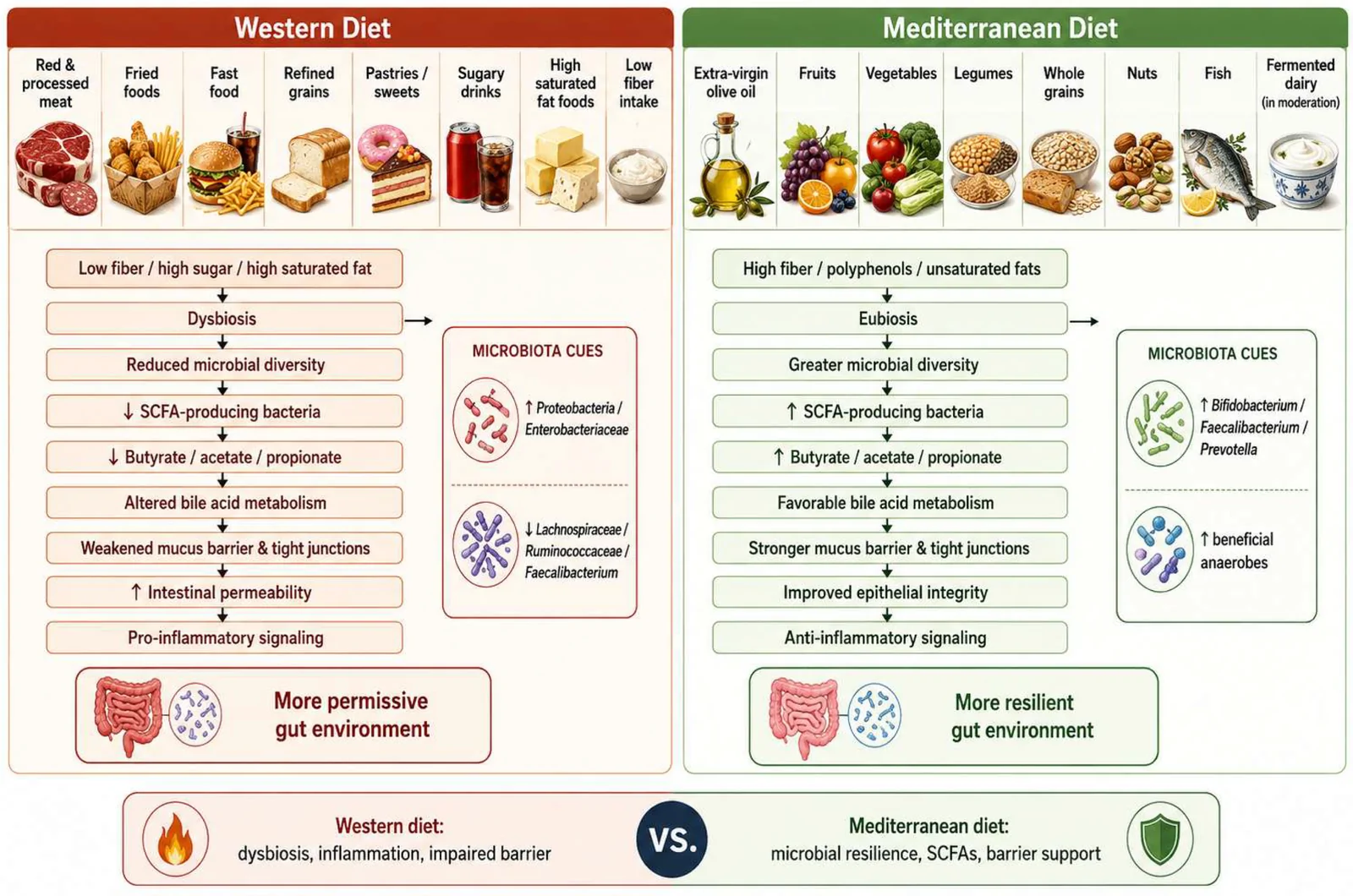
Contrasting effects of Western and Mediterranean dietary patterns on gut microbiota composition, microbial metabolites, and intestinal homeostasis.

Direct evidence on the Mediterranean diet in CDI is still limited, but its biological effects are highly relevant to CDI prevention and recovery. Higher adherence to the Mediterranean diet has been associated with greater microbial diversity, enrichment of fiber-degrading and butyrate-producing taxa such as *Faecalibacterium prausnitzii*, *Bifidobacterium*, *Prevotella*, and beneficial *Clostridium* clusters, as well as increased fecal short-chain fatty acids. These metabolites help maintain epithelial barrier integrity, regulate mucosal immunity, and reduce intestinal inflammation, while plant polyphenols and olive oil–derived bioactive compounds may further promote anti-inflammatory and antimicrobial effects. In contrast to Western diets, which favor dysbiosis, reduced SCFA production, increased permeability, and pro-inflammatory signaling, the Mediterranean diet may help preserve colonization resistance by supporting beneficial anaerobes and microbial metabolic functions involved in bile acid transformation. Although CDI-specific interventional studies are needed, the Mediterranean diet represents a plausible adjunctive nutritional approach to support microbiota recovery, restore protective metabolites, and reduce the intestinal conditions that favor *C. difficile* germination, growth, toxin activity, and recurrence (Figure 2) [71,72,73,74,75,76,77].

Although direct CDI-specific evidence remains limited, the Mediterranean dietary pattern is relevant to this review because it targets several mechanisms involved in colonization resistance, including microbial diversity, SCFA production, bile acid metabolism, epithelial integrity, and inflammation.

### 4.6. Malnutrition, Aging, and Frailty

Malnutrition, aging, and frailty substantially increase vulnerability to CDI and recurrence. Older adults are disproportionately affected because age-related immunosenescence, impaired mucosal defenses, frequent antibiotic exposure, comorbidities, polypharmacy, and repeated contact with hospitals or long-term care facilities all reduce resistance to colonization. Aging is also associated with a less diverse gut microbiota, depletion of protective anaerobes such as *Bacteroides*, *Bifidobacterium*, *Faecalibacterium*, *Roseburia*, and other butyrate-producing taxa, and expansion of potentially pro-inflammatory or opportunistic organisms. These microbial shifts may impair short-chain fatty acid production, weaken epithelial barrier integrity, promote low-grade inflammation, and disrupt bile acid metabolism by reducing the conversion of primary bile acids into secondary bile acids that normally inhibit *C. difficile* growth and toxin activity. Frailty, poor functional status, cognitive impairment, pressure ulcers, and nutritional deficiency have been repeatedly associated with more severe CDI, prolonged symptoms, recurrence, hospital readmission, discharge to long-term care facilities, and increased short- and long-term mortality. In this context, CDI may further amplify the cycle of dysbiosis, inflammation, functional decline, and frailty, making elderly and malnourished patients a particularly high-risk group in whom restoration of microbiota function and bile acid homeostasis may be especially relevant [78,79,80,81].

In elderly and frail patients, nutritional status may therefore influence CDI outcomes by affecting microbiota resilience, epithelial repair, immune competence, and the recovery of bile acid-mediated colonization resistance.

### 4.7. Intensive Care Unit Patients, Artificial Nutrition, and CDI Risk

Critically ill patients are particularly vulnerable to *Clostridioides difficile* infection because several risk factors often coexist in the intensive care unit (ICU), including severe underlying disease, prolonged hospitalization, broad-spectrum antibiotic exposure, impaired mucosal barrier function, altered immunity, invasive procedures, sepsis, and changes in gastrointestinal transit. In this setting, nutrition also differs substantially from habitual oral intake. Many patients receive parenteral nutrition or standardized enteral formulas administered through nasogastric, nasoenteral, or gastrostomy tubes, depending on clinical status and gastrointestinal tolerance [82]. These forms of artificial nutrition may reduce dietary diversity and modify the amount and type of fermentable substrates reaching the colon, with potential consequences for microbial recovery, short-chain fatty acid production, bile acid transformation, and colonization resistance. ICU-associated dysbiosis is characterized by reduced microbial richness and diversity, loss of protective anaerobes such as *Faecalibacterium*, *Blautia*, *Ruminococcus*, *Subdoligranulum*, *Lachnospiraceae*, and *Ruminococcaceae*, and expansion of opportunistic organisms including *Enterococcus* and Enterobacteriaceae. These microbial changes are relevant to CDI because they may impair bile salt deconjugation and 7α-dehydroxylation, reduce secondary bile acid production, and create a luminal environment more permissive to *C. difficile* colonization [83]. Artificial nutrition has also been associated with antibiotic-associated diarrhea in critically ill patients [84]. In the meta-analysis by Wei et al., parenteral nutrition was associated with an increased risk of antibiotic-associated diarrhea, while enteral nutrition was specifically associated with a higher risk of *C. difficile*-associated diarrhea. Patients receiving enteral nutrition may be particularly vulnerable because of prolonged antibiotic exposure, frequent proton pump inhibitor use, exposure to spores through feeding systems, and severe illness-related disruption of the gut ecosystem [85]. However, direct evidence linking specific ICU nutritional regimens to bile acid profiles and CDI outcomes remains limited. Further studies are needed to clarify how artificial nutrition, antibiotic pressure, microbiota disruption, and bile acid metabolism interact in CDI susceptibility and recurrence among critically ill patients [86].

## 5. Therapeutic Implications

### 5.1. Standard CDI Therapies and Limitations

**Figure 3:**
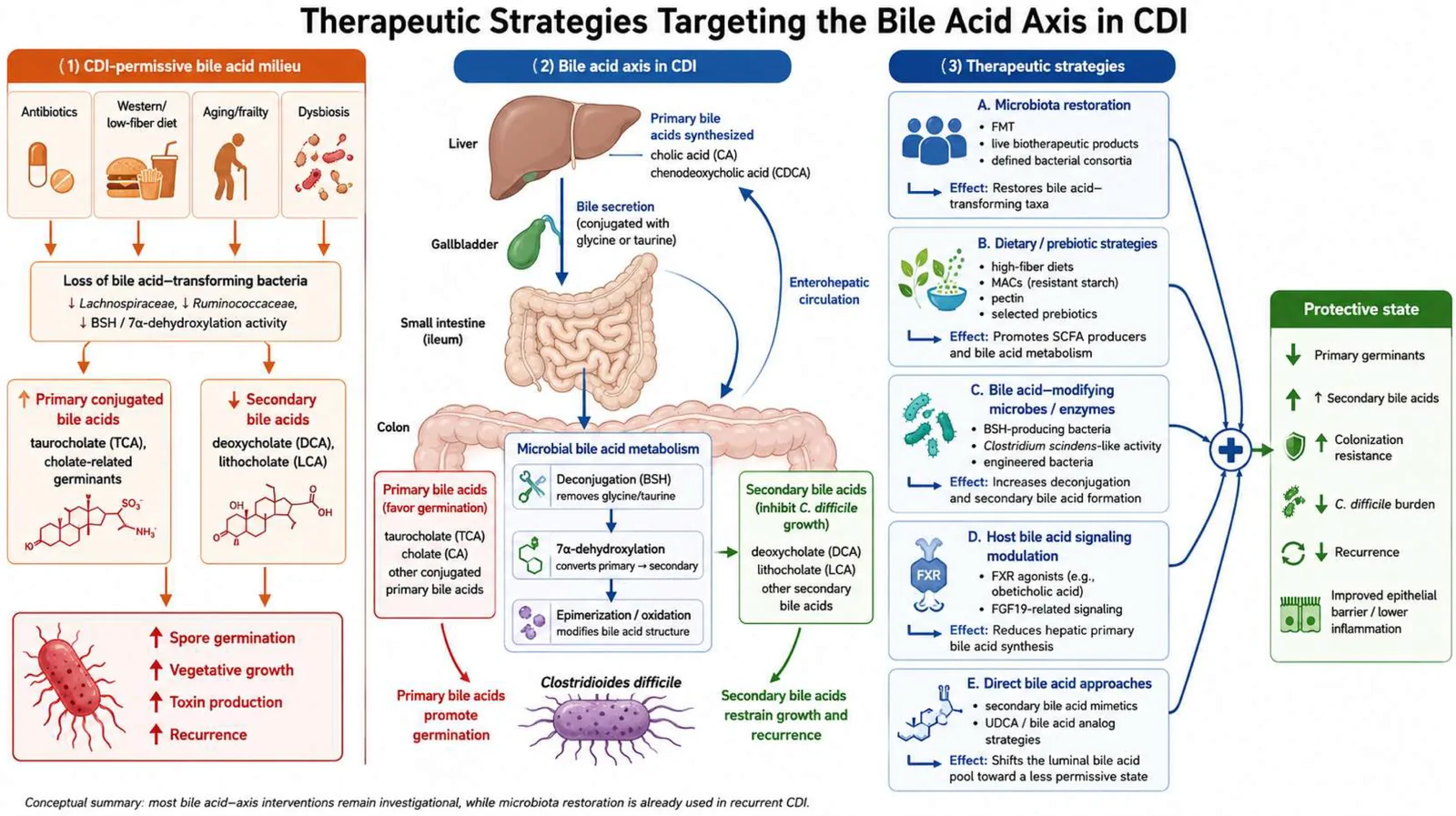
Therapeutic strategies targeting the bile acid axis in *Clostridioides difficile* infection.

Standard treatment for CDI remains centered on antimicrobial therapy, with discontinuation of the inciting antibiotic whenever clinically possible. Fidaxomicin is now preferred for an initial CDI episode because it achieves clinical cure rates comparable to vancomycin while reducing recurrence, likely due to its narrower antimicrobial spectrum and lower impact on the gut microbiota. Oral vancomycin remains an effective and widely used alternative, particularly when fidaxomicin is unavailable or not feasible, whereas metronidazole is no longer recommended as first-line therapy because of lower efficacy, especially in more severe disease. Despite these advances, current therapies have important limitations. Antibiotics suppress *C. difficile* but do not reliably restore the disrupted microbiota, bile acid metabolism, or colonization resistance that underlie susceptibility and recurrence. Fidaxomicin and bezlotoxumab can reduce recurrence in selected high-risk patients, but their use is limited by cost, access, and, for bezlotoxumab, caution in patients with congestive heart failure. Evidence is also limited for optimal management of severe-complicated CDI, as critically ill patients are often excluded from randomized trials, and adjunctive strategies such as intravenous metronidazole, tigecycline, or surgery rely largely on low-quality or observational data. Thus, while standard therapies are effective for controlling acute infection, recurrence, microbiome disruption, treatment accessibility, and limited evidence in severe disease remain major challenges, supporting the need for complementary approaches that restore microbiota function and bile acid homeostasis (Figure 3) [87,88].

These limitations support the need for adjunctive nutritional and microbiome-directed strategies that help restore the metabolic functions, including SCFA production and bile acid transformation, which are not fully recovered by antimicrobial therapy alone.

### 5.2. Fecal Microbiota Transplantation (FMT)

FMT is an established and highly effective therapy for recurrent or refractory CDI, particularly when standard antibiotic treatment fails to restore colonization resistance. By transferring a screened donor microbial community to the recipient, FMT rapidly reshapes the dysbiotic gut microbiota, increasing bacterial richness and restoring taxa commonly depleted in recurrent CDI, including members of *Bacteroidetes*, *Firmicutes*, *Lachnospiraceae*, *Ruminococcaceae*, and *Clostridiales*. One of the main mechanisms proposed for its efficacy is the recovery of microbial bile acid metabolism. Before FMT, patients with recurrent CDI typically show accumulation of primary conjugated bile acids, especially taurocholate, which promotes *C. difficile* germination, together with reduced secondary bile acids such as DCA and LCA, which inhibit vegetative growth and toxin activity. After successful FMT, donor-like microbial functions are restored, including BSH activity, 7α-dehydroxylation pathways, and production of secondary bile acids, thereby creating a less permissive metabolic environment for *C. difficile*. FMT also increases short-chain fatty acid production, particularly butyrate, which supports epithelial barrier integrity, promotes regulatory immune responses, and reduces mucosal inflammation. Although its mechanisms are not limited to bile acids and likely include microbial competition, restoration of SCFA-producing taxa, immune modulation, and possibly effects on the virome and mycobiome, FMT remains a key example of how microbiome-based therapy can correct the metabolic and ecological defects underlying CDI recurrence (Figure 3) [45,89,90,91,92].

The success of FMT also highlights the importance of diet after microbiota restoration, since dietary substrates may influence donor microbial engraftment, secondary bile acid production, and long-term maintenance of colonization resistance.

### 5.3. Microbiome-Based Therapeutics

Microbiome-based therapeutics aim to restore colonization resistance in CDI by correcting the ecological and metabolic deficits left by antibiotic-induced dysbiosis. Beyond conventional FMT, newer strategies include standardized fecal microbiota products, live biotherapeutic products, probiotics, prebiotics, synbiotics, and precision-designed microbial consortia. These approaches seek to reintroduce or stimulate key microbial functions rather than simply suppress *C. difficile* with antibiotics. Their protective mechanisms include restoration of *Bacteroidetes* and *Firmicutes* communities, increased microbial diversity, enhanced competition for nutrients, production of antimicrobial compounds such as bacteriocins, recovery of short-chain fatty acids, and normalization of bile acid metabolism through BSH activity and conversion of primary bile acids into inhibitory secondary bile acids. Commercial microbiota-based products developed for recurrent CDI, including rectal and oral formulations, offer more standardized composition, manufacturing, safety screening, and administration than traditional donor-derived preparations. However, important limitations remain, including variable engraftment, incomplete understanding of the optimal microbial composition, regulatory complexity, cost, access, and limited evidence in severe or fulminant CDI. Overall, microbiome-based therapeutics represent a shift from pathogen-directed treatment toward ecological restoration, but future strategies will likely require more precise, personalized approaches guided by metagenomic, metabolomic, and clinical risk profiles (Figure 3) [93,94,95,96].

Because many microbiome-based therapies depend on the recovery of specific microbial functions, dietary modulation may be necessary to sustain engraftment, metabolic activity, and protection against CDI recurrence.

### 5.4. Bacteriophage Therapy

Bacteriophage therapy represents a potential microbiota-sparing approach for CDI because of its targeted activity against *C. difficile*. Unlike broad-spectrum antibiotics, phage-based strategies could theoretically reduce pathogen burden while preserving commensal taxa involved in bile acid metabolism, short-chain fatty acid production, and colonization resistance. However, clinical translation remains challenging. Many *C. difficile* phages described to date are temperate rather than strictly lytic, raising concerns about prophage integration into bacterial genomes, lysogenic conversion, and potential effects on bacterial fitness, virulence, or toxin regulation. In addition, the narrow host range of many phages may limit activity against the diverse strains involved in CDI, supporting the need for carefully designed phage cocktails with broader coverage [97,98,99,100,101].

Engineered endolysins and recombinant phage-derived enzymes have also gained interest as targeted anti-CDI strategies. These agents may lyse *C. difficile* cells without requiring complete phage replication and may therefore avoid some of the limitations associated with temperate phages. Although most evidence remains preclinical, phage cocktails, engineered endolysins, and other phage-derived antimicrobials may become useful adjunctive strategies, particularly if combined with approaches that restore microbiota function and bile acid-mediated colonization resistance [97,98,99,100,101].

Despite the fact that bacteriophage therapy is not a dietary intervention, it may complement nutrition- and microbiome-based approaches by reducing pathogen burden while preserving microbial functions involved in bile acid metabolism and colonization resistance.

### 5.5. Bile Acid-Targeted Therapies

Bile acid-targeted therapies represent a promising adjunctive strategy for CDI. Therapeutic approaches may aim to reduce the abundance of germination-promoting primary bile acids, particularly taurocholate, increase inhibitory secondary bile acids such as DCA and LCA, or modulate bile acid signaling through pathways including FXR–FGF15/19 and TGR5. Several candidate strategies have been proposed, including bile acid sequestrants, inhibition of enterohepatic bile acid transport, administration of bile acid analogues such as UDCA, and microbiome-directed interventions that restore BSH activity and 7α-dehydroxylation. Experimental data suggest that reducing primary bile acid synthesis or restoring microbial bile acid conversion can decrease *C. difficile* germination and improve disease outcomes, while secondary bile acids may also contribute to epithelial protection and anti-inflammatory signaling. However, most bile acid-targeted interventions remain experimental or indirect in CDI, and their effects may vary according to host metabolism, microbiota composition, enterohepatic circulation, and disease context. Therefore, while modulation of bile acid metabolism is biologically plausible and therapeutically attractive, further clinical studies are needed before these approaches can be integrated into standard CDI management (Figure 3) [102,103,104,105].

These strategies further support the nutritional relevance of CDI management, as diet is one of the major modifiable factors capable of influencing bile acid composition, microbial transformation of bile acids, and host bile acid signaling.

### 5.6. Dietary Interventions

Dietary interventions are emerging as a potential adjunctive strategy in CDI, mainly through their capacity to modulate the gut microbiota, microbial metabolites, epithelial barrier function, inflammation, and bile acid metabolism (Table 3). Patients with CDI, particularly recurrent CDI, typically show reduced microbial richness, depletion of butyrate-producing bacteria such as *Lachnospiraceae* and *Ruminococcaceae*, expansion of non–butyrate-producing Enterobacteriaceae, lower fecal butyrate levels, and reduced microbial capacity for secondary bile acid production. High-fiber diets and microbiota-accessible carbohydrates can promote beneficial anaerobes, increase short-chain fatty acids such as acetate, propionate, and butyrate, strengthen epithelial tight junctions, reduce intestinal permeability, and support anti-inflammatory immune responses. Experimental CDI models suggest that specific fibers or prebiotics, including pectin, inulin, xanthan gum, fructo-oligosaccharides, resistant starches, and complex fermentable carbohydrates, can reduce *C. difficile* burden, improve clearance, and attenuate disease severity, although their effects differ according to fiber type, dose, baseline microbiota, and host condition. Pectin, for example, has shown stronger protective effects than inulin in some models, possibly through improved microbial diversity, increased *Lachnospiraceae* abundance, SCFA production, bile acid modulation, mucosal barrier protection, and reduced inflammatory signaling. Human data remain scarce; no broad dietary intervention trials have been conducted in CDI, although oligofructose supplementation in hospitalized patients was associated with fewer recurrences and increased bifidobacteria despite persistent *C. difficile* carriage. Probiotics, synbiotics, postbiotics, and microbial-derived compounds may also inhibit pathogen growth, modulate toxins, or enhance colonization resistance, but clinical evidence remains inconsistent and safety concerns persist in severely ill or immunocompromised patients. Overall, dietary therapy in CDI is biologically plausible but not yet standardized, and future studies should define which fibers, prebiotics, or whole-diet patterns are most effective for restoring SCFA production, bile acid homeostasis, and microbiota-mediated resistance to recurrence [13,55,97,106,107,108,109].

### 5.7. Future Combination Therapies

Future treatment strategies for CDI are likely to move toward combination approaches that control acute infection while also restoring colonization resistance and preventing recurrence. Antibiotics remain essential for reducing vegetative *C. difficile*, but they do not eliminate spores and may further disrupt the gut microbiota, creating a rationale for adjunctive therapies. Potential combinations include fidaxomicin with bezlotoxumab in patients at high risk of recurrence, microbiome-based interventions such as defined bacterial consortia or microbiota restoration products, and dietary modulation with prebiotics or synbiotics to support beneficial microbial recovery. Probiotics and synbiotics may reduce dysbiosis and inflammation, but their clinical efficacy in CDI remains inconsistent and their use is limited by safety concerns in severely ill or immunocompromised patients. Other emerging strategies include toxin-neutralizing antibodies or proteins, antibiotic neutralizers designed to protect the colonic microbiota, bacteriophage-based therapies targeting specific *C. difficile* strains, non-toxigenic *C. difficile* strains that compete with pathogenic isolates, vaccines, and small molecules that interfere with toxin activity or host inflammatory pathways. Although many of these approaches remain experimental, they highlight a broader therapeutic shift from pathogen suppression alone toward integrated management of CDI through antimicrobial control, microbiota repair, toxin neutralization, immune modulation, and recurrence prevention [115,116,117,118,119].

In this framework, nutrition should be viewed as a supportive component of CDI management, with the potential to enhance microbiota recovery, reinforce bile acid-mediated colonization resistance, and complement antimicrobial or microbiome-based therapies.

## 6. Emerging Research Areas and Knowledge Gaps

Despite growing evidence linking bile acid metabolism, diet, and the gut microbiome to CDI, several important knowledge gaps remain. Much of the current evidence is associative, and further mechanistic studies are needed to clarify whether specific bile acid profiles directly drive CDI susceptibility, recurrence, or recovery, rather than simply reflecting underlying microbiota disruption. Interindividual variability in bile acid metabolism, shaped by diet, age, antibiotic exposure, host genetics, comorbidities, and baseline microbiota composition, may partly explain why patients differ in their risk of CDI and response to microbiome-based or dietary interventions. Multi-omics approaches integrating metagenomics, metabolomics, transcriptomics, and host immune profiling could help identify causal pathways, predictive biomarkers, and patient-specific therapeutic targets. Longitudinal metabolomic studies are particularly needed to track dynamic changes in primary and secondary bile acids before infection, during treatment, and after microbiota recovery. Additional research should also address understudied populations, including pediatric patients, elderly and frail individuals, and patients with recurrent or severe CDI. From a translational perspective, future studies must standardize dietary assessment, intervention design, microbiome analysis, and clinical endpoints to allow meaningful comparison across studies. These advances may support the development of precision microbiome medicine, in which dietary modulation, bile acid-targeted therapies, probiotics, live biotherapeutics, or fecal microbiota-based products are tailored to individual microbial and metabolic profiles.

## 7. Limitations

Several limitations should be acknowledged when interpreting the available evidence. Much of the mechanistic knowledge comes from animal models, which cannot fully reproduce the complexity of human CDI. Differences in gut microbiota composition, bile acid pools, antibiotic exposure, diet, immune responses, and the *C. difficile* strains used may all influence disease severity and treatment outcomes. In addition, CDI is caused by genetically and phenotypically diverse strains, and strain-specific variation may affect sporulation, germination, toxin production, bile acid sensitivity, and response to therapy. The effects of bile acids are also highly context dependent, as individual bile acids may have different, or even opposing, effects depending on their concentration, conjugation status, microbial environment, host factors, and the strain involved. Finally, much of the current human evidence remains associative. Altered bile acid profiles and microbiota changes observed in CDI may contribute to susceptibility and recurrence, but they may also reflect antibiotic-induced dysbiosis, intestinal inflammation, or microbiota recovery after treatment. Future studies combining longitudinal sampling with metagenomics, metabolomics, transcriptomics, and detailed clinical phenotyping are therefore needed to better distinguish causal mechanisms from biomarkers of disease activity, recurrence risk, or microbiota restoration.

## 8. Conclusions

The bile acid–diet–microbiome axis is central to CDI pathogenesis and recurrence. Antibiotic-induced dysbiosis reduces protective secondary bile acids and favors primary bile acids that promote *C. difficile* germination. Diet may further influence CDI risk by shaping microbial diversity, short-chain fatty acid production, bile acid metabolism, barrier function, and inflammation. Future therapies should move beyond pathogen suppression toward restoration of microbiota function and durable colonization resistance.

These bile acids and microbial markers could become biomarkers for stratifying the risk of relapse in clinical practice.

## Figures and Tables

**Table 1:** Primary and secondary bile acids and their effects on *Clostridioides difficile*.

Bile Acid Category	Representative Bile Acids	Main Origin	Effect on *C. difficile*	Biological Relevance in CDI	Refs.
Primary bile acids	Taurocholic acid, glycocholic acid	Synthesized in the liver and conjugated with taurine or glycine	Promote spore germination, especially in the presence of amino acid co-germinants such as glycine	Facilitate the transition from dormant spores to vegetative cells, supporting colonization after microbiota disruption	[29,30,31,32]
	Cholic acid, chenodeoxycholic acid	Produced in the liver; released after microbial deconjugation	Cholic acid derivatives may support germination, whereas chenodeoxycholic acid can inhibit taurocholate-induced germination	The balance between stimulatory and inhibitory primary bile acids influences early CDI susceptibility	
Secondary bile acids	Deoxycholic acid, lithocholic acid	Generated by gut bacteria through 7α-dehydroxylation of primary bile acids	Inhibit vegetative growth and may reduce toxin activity in a concentration- and strain-dependent manner	Contribute to colonization resistance in a healthy microbiota	[34,35,36,37,38,39]
Secondary bile acid derivatives and epimers	Ursodeoxycholic acid, iso-deoxycholic acid, iso-lithocholic acid, 3-oxo-lithocholic acid	Produced through microbial oxidation, epimerization, and other bile acid transformations	May inhibit spore germination, bacterial growth, toxin expression, or toxin-mediated injury, depending on the compound	Reflect the protective metabolic activity of a diverse gut microbiota	[34,35,36,37,38,39]

CDI = *Clostridioides difficile* infection; Refs. = references.

**Table 2:** Protective and non-protective dietary patterns in experimental models of *Clostridioides difficile* infection.

Dietary Role in CDI	Interpretive Category	Dietary Strategy Evaluated	Antibiotic Exposure	Main CDI-Related Finding	Ref.
Protective effect of higher fiber intake	Fiber enrichment/microbiota-accessible carbohydrates	Low- versus high-fiber diets	Kanamycin, gentamicin, colistin, metronidazole, vancomycin, clindamycin	Low fiber was associated with more severe disease and slower recovery, whereas high fiber reduced disease severity and accelerated recovery.	[54]
Protective effect depending on fiber type	Fiber type-specific effects	Pectin- or inulin-rich diets compared with cellulose	Kanamycin, gentamicin, colistin, metronidazole, vancomycin, clindamycin	Pectin increased microbiome diversity and lowered inflammatory markers more effectively than inulin or cellulose.	[55]
Protective effect of microbiota-accessible carbohydrates	Microbiota-accessible carbohydrates and SCFA production	Seven diets supplemented with distinct MACs	Clindamycin	*C. difficile* burden was significantly reduced after MAC supplementation, particularly when cecal butyrate levels were highest.	[56]
Protective effect of xanthan gum supplementation	Specific fiber supplementation	Standard chow supplemented with 5% xanthan gum	Cefoperazone or kanamycin, gentamicin, co-listin, metronidazole, vancomycin, clindamycin	Xanthan gum supplementation limited microbiome disruption and reduced susceptibility to *C. difficile* colonization.	[57]
Non-protective effect of a high-fat/low-fiber pattern	Western-type high-fat/low-fiber pattern	High-fat/low-fiber diet compared with chow or low-fat/low-fiber diet	Kanamycin, gentamicin, colistin, metronidazole, vancomycin, clindamycin	The high-fat/low-fiber diet was associated with worse CDI-related outcomes than chow or low-fat/low-fiber diets.	[58]
Non-protective effect of a high-fat/high-protein pattern	High-fat/high-protein macronutrient pattern	High-fat/high-protein diet compared with high-fat/low-protein or high-carbohydrate diet	Kanamycin, gentamicin, colistin, metronidazole, vancomycin, clindamycin	Mortality was higher in mice receiving the high-fat/high-protein diet than in those receiving the high-fat/low-protein diet.	[59]
Non-protective effect of high-fat feeding in obesity-associated CDI	High-fat diet in obesity-associated CDI	High-fat diet in obese mice	Cefoperazone	High-fat feeding impaired *C. difficile* clearance and increased clinical manifestations, including diarrhea and weight loss.	[60]
Non-protective effect of a high-carbohydrate diet in a hamster model	High-carbohydrate diet in hamster CDI model	High-carbohydrate diet compared with chow	Clindamycin	High-carbohydrate feeding was associated with higher mortality and persistent dysbiosis compared with chow.	[61]

CDI = *Clostridioides difficile* infection; MAC = microbiota-accessible carbohydrates; SCFA = short-chain fatty acids; Ref. = references.

**Table 3:** Nutritional strategies with potential relevance to microbiota recovery and colonization resistance in CDI.

Therapeutic Direction	Dietary Approach	Key Nutritional Features	Microbiota-Related Rationale	Expected Benefit	Ref.
Support beneficial microbial fermentation	Fiber-rich dietary pattern	Emphasizes fruits, vegetables, legumes, and whole grains as major sources of dietary fiber	Provides fermentable substrates that favor beneficial bacteria and support SCFA-producing communities important for intestinal health	Supports SCFA production, epithelial barrier integrity, and restoration of colonization resistance after antibiotic-induced dysbiosis.	[110]
Restore microbial balance through fermented foods	Probiotics and fermented foods	Includes yogurt, kefir, sauerkraut, kimchi, and other probiotic-rich or fermented foods	Helps restore microbial balance and may improve microbiota composition when consumed adequately	May contribute to microbial recovery, although CDI-specific efficacy remains inconsistent and safety should be considered in vulnerable patients.	[111]
Limit dietary factors that favor dysbiosis	Restriction of added sugars and saturated fats	Reduces intake of foods high in added sugars and saturated fats	May restrict the expansion of pathogenic bacteria and reduce diet-associated intestinal inflammation	May reduce diet-associated dysbiosis, intestinal inflammation, and microbial patterns that weaken colonization resistance.	[112]
Selectively nourish beneficial bacteria	Prebiotic-rich foods	Includes garlic, onions, Jerusalem artichokes, and non-digestible fiber-rich vegetables	Prebiotics provide substrates that selectively promote beneficial bacteria in the colon	Selectively supports beneficial fermentative bacteria and may promote SCFA-mediated protection against *C. difficile* expansion.	[113]
Promote dietary diversity and bioactive nutrient intake	Mediterranean-style diet	Emphasizes fruits, vegetables, fish, whole grains, legumes, nuts, and vegetable oils; includes moderate dairy and poultry and limited red meat and processed foods	Provides diverse nutrients and bioactive compounds that support microbiota diversity and intestinal health	May support microbial diversity, butyrate-producing taxa, anti-inflammatory metabolites, and bile acid transformation relevant to CDI prevention or recovery.	[114]

Ref. = references.

## Data Availability

This review is based exclusively on data from previously published studies, all of which are cited in the reference list. No new datasets were generated or analyzed during the current study.
